# How do health services engage culturally and linguistically diverse consumers? An analysis of consumer engagement frameworks in Australia

**DOI:** 10.1111/hex.13315

**Published:** 2021-07-15

**Authors:** Ashfaq Chauhan, Ramesh L. Walpola, Elizabeth Manias, Holly Seale, Merrilyn Walton, Carlene Wilson, Allan B. Smith, Jiadai Li, Reema Harrison

**Affiliations:** ^1^ School of Population Health UNSW Sydney Kensington New South Wales Australia; ^2^ School of Nursing and Midwifery, Centre for Quality and Patient Safety Research, Institute for Health Transformation Deakin University Melbourne Victoria Australia‎; ^3^ School of Public Health The University of Sydney Sydney New South Wales Australia; ^4^ Olivia Newton‐John Cancer Wellness and Research Centre Austin Health Heidelberg Victoria Australia; ^5^ School of Psychology and Public Health La Trobe University Bundoora Victoria Australia; ^6^ Centre for Oncology Education and Research Translation (CONSORT), Ingham Institute for Applied Medical Research University of New South Wales Liverpool New South Wales Australia

**Keywords:** consumer engagement, document analysis, engagement frameworks, ethnic minority, health policy, patient participation

## Abstract

**Background:**

Engagement frameworks provide the conceptual structure for consumer engagement in healthcare decision making, but the level to which these frameworks support culturally and linguistically diverse (CALD) consumer engagement is not known.

**Objective:**

This study aimed to investigate how consumer engagement is conceptualised and operationalized and to determine the implications of current consumer engagement frameworks for engagement with CALD consumers.

**Method:**

Altheide's document analysis approach was used to guide a systematic search, selection and analytic process. Australian Government health department websites were searched for eligible publicly available engagement frameworks. A narrative synthesis was conducted.

**Results:**

Eleven engagement frameworks published between 2007 and 2019 were identified and analysed. Only four frameworks discussed engagement with CALD consumers distinctly. Organisational prerequisites to enhance engagement opportunities and approaches to enable activities of engagement were highlighted to improve CALD consumers' active participation in decision making; however, these largely focused on language, with limited exploration of culturally sensitive services.

**Conclusion:**

There is limited discussion of what culturally sensitive services look like and what resources are needed to enhance CALD consumer engagement in high‐level decision making. Health services and policy makers can enhance opportunities for engagement with CALD consumers by being flexible in their approach, implementing policies for reimbursement for participation and evaluating and adapting the activities of engagement in collaboration with CALD consumers.

**Patient/Public Contribution:**

This study is part of a wider ‘CanEngage’ project, which includes a consumer investigator, and is supported by a consumer advisory group. The study was conceived with inputs from the consumer advisory group, which continued to meet regularly with the project team to discuss the methodology and emerging findings.

## 1 INTRODUCTION

Contemporary healthcare systems identify consumer engagement as a cornerstone for patient‐centred, value‐based care.^1^, ^2^, ^3^ Consumers are broadly defined as patients, families, carers and communities who are current, past or potential users of health services.^4^ Consumer engagement is the active participation of consumers in the decision‐making process to improve healthcare, and it occurs on a spectrum from information provision through to consumer‐led decision making about systems and services of care.^3^ Effective consumer engagement has many benefits, including higher satisfaction amongst clinicians and consumers with the care provided, improved resource allocation, the cost‐effectiveness of service delivery, targeted therapeutic initiatives and interventions for consumers and identification of opportunities to innovate for better care quality and health outcomes.^2^, ^5^, ^6^, ^7^, ^8^ Patient‐centred care involves asking patients what matters to them most and being respectful of their preferences, needs and values.^9^ Consumer engagement intersects with patient‐centred care; they are mutually grounded in trust, respect, shared knowledge and a positive relationship between patients, carers and health professionals.^9^, ^10^

Central to realising the benefits of consumer engagement is ensuring ease of consumer–provider interaction, high levels of consumer health literacy, support and guidance regarding the engagement activities and efforts towards even power distribution between consumers and health professionals.^11^, ^12^, ^13^ As such, effective consumer engagement for culturally and linguistically diverse (CALD) consumers is close to nonexistent.^14^ CALD is primarily used as a term in the Australian context to describe those who were born overseas, have parents who were born overseas or speak languages other than the official national languages and/or have lower proficiency of native or national languages.^15^ Inequities in engagement identified in these communities are further compounded by fears of intimidation, low self‐confidence in their own healthcare, racism, gender inequalities, sex, disabilities, low trust in health professionals and the health care system and communication barriers.^16^, ^17^, ^18^ A 2018 survey study in Australia examining activities for consumer participation as indicated by the National Safety and Quality Health Services (NSQHS) Standards found that only 50 out of 115 Australian health services reported including people of CALD background in decision‐making processes.^19^

There is emerging evidence identifying factors that present barriers to CALD consumer engagement, leading to variations in access to and experience of healthcare, and poorer health and healthcare quality outcomes.^14^, ^20^, ^21^, ^22^ A recent systematic review concluded that safety events (events that could have or did result in harm due to care they receive) among CALD patients are dependent on the setting and the population, and that poor engagement with CALD consumers is one factor associated with their increased vulnerability to safety events.^23^ Poor engagement is often associated with an inability of the health system to recognise and address the nuanced sociocultural differences that exist between and within diverse CALD groups and with a lack of responsiveness to those differences.^14^, ^23^ In multicultural Australia, where over 300 languages are spoken and almost half of the Australians are born overseas or had one or both parents born overseas,^15^ it is imperative for health systems and services to provide a context that facilitates effective engagement for CALD consumers towards improved care quality and outcomes.

The importance for consumer engagement in healthcare is constructed to a large extent through health systems policies, standards and guidance for consumer engagement that have emerged over the last two decades nationally and internationally.^24^, ^25^, ^26^, ^27^, ^28^, ^29^ These documents highlight the increasing importance of consumer engagement in healthcare and provide policy directives, guidance and avenues to integrate consumer participation in decision making. Consumer engagement frameworks provide a conceptual structure for engaging consumers in decision‐making processes at various levels of the healthcare system.^3^ The narrative of such frameworks provides a context for consumer engagement that is relevant to the populations being served and to system responsiveness to minority and/or priority populations such as CALD communities.^3^, ^30^, ^31^ Engagement frameworks adopted by Australian federal‐ and state‐level government health departments provide direction and understanding of an organisation's commitment to consumer engagement to operationalise health system goals at individual, service and system levels. These frameworks also provide a reference point for accountability and promote leaders, managers and clinical teams to be more responsive to consumers, and allocate appropriate resources for engagement.^3^, ^11^, ^32^ The extent to which these frameworks articulate engagement activities and recognise sociocultural differences for CALD consumers is unknown. This knowledge is critical to inform and ensure a health system context that considers and is responsive to the CALD population.

The aim of this document analysis of consumer engagement frameworks in Australia at federal‐ and state‐level health departments was to examine the health system narrative regarding CALD consumer engagement and the extent to which these frameworks may promote and support nuanced approaches for engagement with the CALD population. This study aims to explore: (1) how consumer engagement is conceptualised across the Australian healthcare system; (2) how consumer engagement is operationalized; and (3) the implications of current consumer engagement frameworks for engagement with CALD consumers.

## 2 METHODS

A document analysis utilising a systematic process for the search, selection and analysis was undertaken to address the research aims.^33^, ^34^ This widely adopted approach has been used to explore policy positioning in healthcare settings.^35^, ^36^ We used Altheide's document analysis approach to guide the research process, due to its effective application in similar work.^34^, ^36^ The Standard for Reporting Qualitative Research guideline was used for reporting this paper^37^ (Supporting Information File SA).

### 2.1 Document search and selection

Document selection was undertaken through an iterative search and selection process. An initial document search of government websites was conducted between June and July 2020 by two reviewers (A. C. and J. L.) using various key search words (consumer/patient engagement, consumer/patient engagement framework, consumer/patient participation, consumer/patient involvement). This search identified an initial set of 40 documents (engagement frameworks, strategic plans, consumer engagement guidelines, consumer engagement strategies, consumer engagement toolkits and cultural diversity plans). These 40 documents were then discussed with the last reviewer (R. H.) to further refine the search strategy and to develop eligibility criteria.

### 2.2 Eligibility criteria

*Eligible data sources*: Federal‐, state‐ and territory‐level government health departments and the associated agencies were included as they are primarily responsible for setting principles and policies for the delivery of health services in Australia. Engagement frameworks originating between January 2001 and July 2020 were included. This time period was selected because consumer engagement has increasingly been prioritised in the last 20 years.

*Types of documents*: We defined consumer engagement frameworks as documents that provided a conceptual structure for engagement with consumers in the decision‐making process at various levels of health systems, and that outlined levels and the spectrum of engagement, and the methods and activities of engagement.

Data sources and documents that did not fulfil the above criteria were excluded. Individual local health district‐level consumer engagement frameworks were excluded as they linked up to higher‐level frameworks included in the analysis. Consumer engagement toolkits, guidelines and implementation plans were excluded as these were outside of the definition. Engagement frameworks for mental health services or drug and alcohol services were also excluded as these were considered very specific areas of engagement for engaging with other minority and priority population groups and were not relevant to the study objectives.

*Unit of analysis*: The aspects of the framework that discussed the concept of engagement, principles of engagement, process of engagement, type of engagement and activities of engagement with consumers were analysed along with how CALD populations are represented in these documents and what special considerations were made for CALD consumer engagement.

### 2.3 Document collection and data extraction

After establishing the eligibility criteria, the same reviewers (A. C. and J. L.) applied this to the initial set of 40 documents identified, along with another search conducted of the Australian Government (federal, state and territory) health department websites and associated agencies for eligible documents (see Supporting Information File SB for the full list of the website searched). This search identified 11 publicly available engagement frameworks that fulfilled the eligibility criteria. A protocol for data extraction was developed based on the research aims. Data were independently extracted by the same reviewers (A. C. and J. L.) under the following categories: federal or state level; health system or service level; organisation, document title and year of publication; purpose of the document; key messages regarding consumer engagement; and diverse populations discussed (Table 1).

**Table 1:** Data extraction table

Organisation, title	State/federal system/service/individual	Purpose of the document	Key messages for consumer engagement	Type of diverse population/group discussed and specific CALD considerations
Queensland Health	State level	This document provides a framework for effective consumer and community engagement for health services organisations	Definition of a consumer, carer, consumer engagement and community engagement are provided at the outset	An example is provided for a network‐level engagement for local health and hospital networks to partner with multicultural organisations within the network to understand the need of the population
Consumer and Community Engagement Framework (2012)	Service/organisational level	This document is designed to guide the LHHNs strategies for consumer and community engagement in QLD	Based on the IAP2 framework	No specific discussion on CALD groups included
			Framework is supported by *nine overarching principles* that supports engagement: (1) participation; (2) person‐centred; (3) accessible and inclusive; (4) partnership; (5) *diversity*; (6) mutual respect and value; (7) support; (8) influence; and (9) continuous improvement	
Department of Health, WA	State level	The document is developed to assist health staff, area health services and WA health in implementing effective engagement with consumers	The framework is founded on the four levels of consumer, carer and community engagement: (1) individual client or patient interaction; (2) department, programme or service level; (3) area health service level; and (4) WA health level	Recognises that some groups are disproportionately involved in consumer engagement processes
WA Health Consumer Carer and Community Engagement Framework: for health services, hospitals and WA Health following consultation across WA Health (2007)	Service/organisational & system level	This framework provides an action plan for embedding consumer engagement as an activity will be embedded as a core activity.	Range of consumer participation varies from low to high level of control from none to receive information, is consulted, advices organisation, plans jointly, has delegated control, has control	No specific strategies for CALD engagement proposed
			Framework is based on nine principles of consumer engagement: (1) trust; (2) respect; (3) openness; (4) equal opportunity; (5) advocacy and support; (6) responsiveness; (7) shared ownership and accountability; (8) dissemination; and (9) evaluation	
			Provides definition of consumers, carers and community at the outset	
Safer Care Victoria, VIC	State level	Bring consistency to how Victorians can participate in their healthcare	Definition of a consumer is included	Recognises diversity and provides a special section for CALD stakeholders within the document
Partnering in Healthcare (2019)	Service/organisational level	Help health services deliver care that is safe, person‐centred, family‐centred, equitable and clinically effective	Partnering in a healthcare framework consists of five domains: (1) Personalised and holistic; (2) working together; (3) shared decision making; (4) equity and inclusion; and (5) effective communication	
		Clearly describe consumer priorities for health services, Safer Care Vic and Department of Health and Human Services (DHHS)		
DHHS, VIC	State level	Provide a consistent understanding of expectations, roles and responsibilities of staff when engaging with stakeholders and undertaking public participation activities	Definitions of stakeholder, stakeholder engagement, public engagement, public participation, codesign and human‐centred design are provided	Recognises diversity and provides a special section for CALD stakeholders within the document
Public participation and stakeholder engagement framework (2019)	System and service/organisation Level	Act as a consolidated reference point for staff, providing an overview of endorsed stakeholder engagement and public participation methodology and support	Five engagement principles informing the framework: (1) Purposeful; prepared; genuine; inclusive; and communicate	
			The framework is based on the IAP2 framework. The spectrum helps define the five potential roles for stakeholders in any engagement process: inform, consult, involve, collaborate and empower	
			Outlines the six key elements used to audit the efficiency and effectiveness of public participation activities. These elements are aligned with—and support the implementation of—our engagement principles	
Department of Health, Tasmania Government	State level	To guide the Tasmania Health Organisation (THO) to effectively engage with consumers in planning, delivery and evaluation of care	based on the IAP2 framework of engagement from inform to consult, involve, partner and delegate	The CALD population is discussed in the context of communication barriers
Consumer and Community Engagement Framework 2015‐2018 (2015)	System/organisation level	To consolidate and extend all collaborative, integrated and effective engagement initiatives into a comprehensive framework and to describe an agreed direction for partnering with consumers in the THO‐South	Consumer and community engagement will operate across four levels: individual; communities; service; and the DHHS System	No other specific discussion of CALD consumer engagement strategies
			Underpinning principles to support and implement engagement framework are (1) participate; (2) dissemination; (3) consumer‐centred; (4) diversity; (5) respect; (6) training and support; and (7) continuous Improvement	
			Includes the definition of a consumer, Community, involvement, participation and patient‐centred care	
South Australia Health	State level	It is written for all SA Health employees including local health networks (divisions, hospitals, wards, departments, service and primary health services and central office divisions)	Definition of a consumer, consumer engagement, partnership, community, community engagement, consultation, patient and consumer‐centred care, health literacy, etc. is provided	The only mention of any diversity (CALD population) is in the principles on which the framework is to be implemented
A Framework for Active Partnership with Consumers and the Community (2013)	Services and health professionals		The principles are (1) partnership; (2) engagement; (3) patient‐ and consumer‐centred care; (4) diversity; (5) feedback and consumer experience; (6) empower consumers and the community to be equal partners in care and treatment; (7) access and information; (8) support; (9) charter of Health and Community Service rights; (10) continuous improvement, measuring and evaluation; and (11) consumers and the community, and research and evaluation	No specific discussion of how to achieve engagement with CALD consumers
			No levels or spectrum of engagement are discussed in this framework	
			Sets out the standards that are in line with the NSQHS standards that the framework aims to accomplish	
Northern Territory Department of Health	State level	Rational presented, but the purpose is unclear	Definition of stakeholder engagement is provided, and incorporates citizen and consumer engagement, public or community participation	Diversity discussed in the context of inclusion principle— when there is an opportunity for a diverse range of values and perspectives to be freely and fairly expressed and heard
Stakeholder Engagement Framework (2012)	System/service level		Spectrum of engagement is based on the IAP2 methodology and incorporates spectrum of engagement encompassing from inform to consult, involve, collaborate and empower	
			Stakeholder engagement is based on the principles of *integrity, inclusion, deliberation and influence*	
Cancer Institute NSW	State level	Developed in line with the national framework for consumer involvement in cancer control	Provides the definition of consumer and community	No discussion of CALD consumers specifically
Consumer and Community Engagement Framework (2015)	Service level	While there are many examples of successful consumer engagement across the Cancer Institute NSW, implementing the framework aims to move this beyond ‘committee representation’ to incorporate the five levels of participation outlined in the National Framework for Consumer Involvement in Cancer Control	Integrates the elements for effective consumer involvement, as outlined in the National Framework for Consumer Involvement in Cancer Control: (1) committed organisations; (2) capable consumers; (3) inclusive groups; and shared focus	
			Five levels of participation outlined are: engage and inform; consult; involve; partnership; and consumer led	
NSW Agency for Clinical Innovation (ACI)	State level	Alignment with ACSQHC Standard 2	Level of engagement based on the IAP2 framework	CALD consumers identified by partners including multicultural health manager and CALD statewide services
Patient Experience and Consumer Engagement: A Framework for Action (2015)	ACI and partnering health services in NSW Health	Provide a way to incorporate consumers in care delivery redesign across NSW	Five levels of participation outlined are engage and inform; consult; involve; partnership; and consumer led	
			Development of PEACE teams to effectively engage consumers in health innovation in NSW	
Department of Health, Australia	Federal level	Outlines key actions and capability development agenda and strategic priorities	Level of engagement based on the IAP2 framework	Specific section on CALD consumer engagement
Stakeholder engagement framework			Outlines the process of engagement with evaluation as key element	Emphasised need for developing culturally sensitive services
			Principles for engagement are outlined: (1) purposeful; (2) inclusive; (3) timely; (4) transparent; and (5) respectful	
Cancer Australia	Federal level	Promote and support organisations committed to involving consumers in cancer control	The Framework identifies the needs of consumers participating in cancer control and the expectations of health professionals, service managers, researchers and policy makers who seek to engage consumers successfully	CALD discussed along with other minority and priority groups
National Framework for Consumer Involvement in Cancer Control		Provide principles to govern consumer engagement	Level of engagement based on background paper on community engagement prepared for NICE	
			Outline organisational and individual capacity as important steps towards successful consumer engagement	

Abbreviations: ACSQHC, Australian Commission on Safety and Quality in Health Care; CALD, culturally and linguistically diverse; IALP2, International Association of Public Participation; LHHN, Local Health and Hospital Networks; QLD, Queensland; NICE, National Institute of Health and Care Excellence; NSQHS, National Safety and Quality Health Services; and NSW, New South Wales.

### 2.4 Data analysis and reporting

Arnstein's Ladder of citizen engagement^38^ and Carman et al's.^3^ frameworks for patient and family engagement were used to guide the narrative data synthesis. These two frameworks are well‐established engagement frameworks used to differentiate between tokenistic versus meaningful engagement across the continuum of consumer involvement in the decision‐making process and hence were considered relevant for this document analysis.^3^, ^38^, ^39^ Along with the original data extraction tool (Table 1), a separate table (Supporting Information File SC) was developed drawing on the similarities and differences between the selected frameworks, focusing on elements of conceptualisation and operationalization of these frameworks. The data extracted were then explored to identify what support structures and contextual features were proposed or adopted by health services in Australia to engage with consumers and how these were discussed in the context of CALD consumers.

A narrative synthesis was conducted. Synthesising data using a narrative synthesis approach allowed us to establish a relationship between research, policy and practice and generate key common concepts relevant to the research objectives.^40^, ^41^, ^42^ Key information from each set of documents was extracted on a data extraction form (Table 1) to provide a narrative on common themes. AC conducted the initial analysis of the data. Data were grouped into categories resembling the research questions and common narratives across the documents examined and analysed. Following this grouping, the findings were discussed with the wider research team and the analysis was further refined. Any disagreements and differences were resolved with discussion.

## 3 RESULTS

### 3.1 Data sources and characteristics

**Figure 1:**
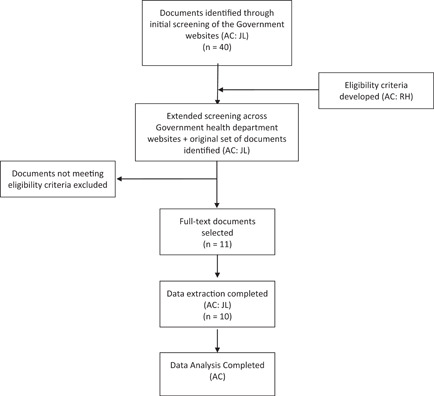
Flow chart for document search and selection

Using Altheide's document analysis approach (Figure 1), a total of 11 engagement frameworks, two at the federal level and nine others across the state and territory levels, were identified as eligible.^43^, ^44^, ^45^, ^46^, ^47^, ^48^, ^49^, ^50^, ^51^, ^52^, ^53^ Of the eleven frameworks, three were identified as stakeholder engagement frameworks, while eight comprised community and/or consumer engagement. Where the term stakeholder was used, the term was described as inclusive of consumers and community. The documents were published between 2007 and 2019, with two engagement frameworks scheduled for review in 2018 and 2019, but updated versions of the documents were not publicly available at the time when the search was completed.^44^, ^51^ Two documents were identified as living documents and subject to regular updates.^46^, ^47^ All frameworks were directed at the organisational level with the purpose of facilitating engagement with consumers to improve health outcomes, and to comply with Standard 2: Partnering with Consumers Standard of the NSQHS Standards.^25^ This standard was implemented in 2012, recognising the role of consumers as partners in planning, delivery, measurement and evaluation of systems and services and in planning their own care.^25^ Two documents were cancer‐specific, outlining engagement with consumers in cancer services to improve health outcomes.^43^, ^53^

### 3.2 Q.1) How is engagement conceptualised?

Nine frameworks provided an explicit definition for consumer engagement.^44^, ^45^, ^46^, ^47^, ^49^, ^50^, ^51^, ^52^, ^53^ Engagement in this definition was expressed as the process by which consumers are involved in decision making. This process is consistent with the Deming management approach to continuous improvement of the Plan, Do, Study and Act Cycle.^54^ The process of engagement was further conceptualised in three frameworks as an ongoing cycle supporting engagement activities via thorough planning, preparation, action and evaluation of the activities.^45^, ^47^, ^48^ Involvement in the process was articulated as either participation in the whole process or participation in specific activities within the process.^45^, ^47^, ^48^ A wide range of concepts were included that described the nature of activities, ranging from consultation through to participation and codesign, reflecting engagement as a continuum. The term ‘participation’ was also used as a synonym for engagement rather than a specific level of engagement in two documents.^46^, ^47^

Principles for engagement were explicitly mentioned in eight frameworks,^44^, ^47^, ^48^, ^49^, ^50^, ^51^, ^52^, ^53^ with the four most common principles identified as (1) being purposeful: (2) being participatory in nature; (3) being inclusive and diverse; and (4) continuous improving. These categories closely resembled the process of engagement (planning, preparing, action and evaluation). Being purposeful was described as staying committed to the task and having a clear expectation and understanding of the task, activities and outcomes between health services and the consumers.^48^ Being participatory involved working together with consumers in a partnership, with shared ownership and accountability of the process of engagement.^47^ Being inclusive and diverse was proposed to promote equity within the engagement process and support diverse consumer participation in the process. Being inclusive and diverse varied from being inclusive as much as possible to the ability to embrace diversity where relevant^44^ to identifying opportunities and working collaboratively with a broad range of consumers.^49^ Continuous improvement focused on evaluating engagement activities to improve future opportunities for engagement. This principle also included the dissemination of results to collect feedback from consumers and for quality assurance.^44^, ^48^, ^49^, ^50^

### 3.3 Q.2) How do consumer engagement frameworks operationalise engagement?

Each document was explored with regard to the operationalization of consumer engagement. Information was examined about the elements of effective engagement, sources for the model of engagement discussed in each framework and the suggested methods and activities proposed to realise the engagement. Key elements required in a service to promote effective engagement were identified as building capacity amongst consumers and organisational staff, having a clear description of the proposed level of participation in a given activity, having financial and physical resources for participation and dedicated time for each task and providing information to consumers for their review.^43^, ^44^, ^46^, ^47^, ^48^, ^49^, ^50^, ^53^ Underpinning organisational features were also identified as prerequisites to successful engagement such as an organisational commitment to engagement,^43^, ^47^, ^53^ where diverse consumers participate in advisory committees,^44^, ^45^, ^46^, ^47^, ^48^, ^49^, ^50^ within a culture of engagement without intimidation, promoting the free exchange of information and positive relationships between consumers and services.^44^, ^45^, ^46^, ^47^, ^48^, ^49^, ^50^, ^51^, ^52^ Evaluation of engagement was discussed in 10 frameworks^44^, ^45^, ^46^, ^47^, ^48^, ^49^, ^50^, ^51^, ^52^, ^53^ with 7 presenting information on methods of evaluation^44^, ^45^, ^46^, ^47^, ^49^, ^50^, ^52^; however, from a consumer engagement point of view, the method of evaluation appears to have been largely limited to collecting feedback from consumers using surveys.

Although active and meaningful participation in activities was emphasised as critical; one framework at the federal level highlighted that this participation depends on the purpose, task, roles and responsibility, and the issue being addressed.^48^ Models of engagement proposed in the frameworks were adopted from various sources, such as the International Association of Public Participation (IAP2); NSQHS Standard 2: Partnering with consumers; the National Institute of Health and Care Excellence (NICE) framework for community engagement; and the Brager and Specht participation continuum. The nature of engagement proposed and the activities or methods relevant to the nature of engagement were outlined in eight frameworks.^43^, ^44^, ^45^, ^47^, ^48^, ^49^, ^50^, ^53^ The frameworks described engagement across the various levels of involvement in the decision‐making process, depending on the different models used. The frameworks that used the IAP2 model, NSQHS Standard 2: Partnering with consumers and the NICE framework for community engagement, outlined the level of engagement as participation continuum across five stages (inform, consult, involve, collaborate/participate and empower/consumer‐led).^43^, ^44^, ^47^, ^48^, ^50^, ^53^ The framework that used the Brager and Specht model outlined the level of engagement across seven stages of consumer control in decision‐making (none, received information, is consulted, advises organisation, plan jointly, has delegated control and has control).^52^ Although various stages were used to describe the level of engagement, similarities existed between their classifications. Based on these similarities, the level of involvement can be categorised into three main areas resembling the categories proposed by Carman et al.^3^: (1) information sharing; (2) collaboration in decision making; and (3) shared partnership and leadership. These areas represented the continuum of engagement from low to high levels of involvement and decision‐making power. No explicit involvement in decision making was proposed in the first category, while the second and third categories proposed an explicit role in decision making, with the delegation for decision making provided to consumers in the last category.

Various activities and methods of engagement were suggested across the continuum of engagement. These included display units and surveys for information sharing; focus groups and workshops for collaborations; and consumer participation in quality and safety committees for consumer lead approaches.^47^, ^48^, ^49^, ^50^ These activities were discussed in the context of level of participation needed from consumers. There was a lack of discussion and clarity on the relationship between the activities used and intended outcomes. Table 2 presents an overview of suggested engagement methods and activities included in the various engagement frameworks along with the most common activities highlighted.

**Table 2:** Proposed methods and activities for engagement across the engagement frameworks

Information sharing (no participation in decision making)		Collaboration (participation in decision making)		Shared participation and leadership in decision making
Inform	Consult	Involve	Collaborate	Empower/control
Displays (1–5)	Surveys (1–8)	Advisory panels (2, 5, 8)	Advisory committees (1, 3–7)	Committees: quality, steering (1–3, 5, 7)
Fact sheets (1–4, 6, 7)	Focus groups (1–3, 5–8)	Workshops (1–4, 7)	Participatory decision making (1, 5, 8)	Boards (1, 3, 5, 7)
Media releases (1–4, 6)	Public meetings (2–4, 6, 7)	Working parties (1, 3, 5–7)	Working parties (1, 5, 8)	Standing strategic committees (2, 3, 7)
Websites (1–7)	Workshops (2, 5, 6, 8)	Consumer representation on management committees (4, 5, 8)	Clinical networks (3, 7)	Citizen jury (1, 6)
Letters (2, 4, 6)	Conferences (3, 4)	Forums (2, 4, 6, 7)	Citizen jury (1)	Community‐appointed management committees (4)
Flyers (4)	Forums (4, 8)	Panels (2, 3, 7)	Expert advisory panel (6)	Multipurpose health services (3)
Posters (6)	One‐on‐one meetings (2)	Conferences (1, 3)	Facilitated consensus building (7)	Participatory budgeting (6)
Public meetings (4, 7)	Consumer representation on management committees (4)	Consultative committees (2, 6)	Hackathon (6)	Participatory governance (5, 7)
Advertising (4)	Discussion papers (1, 3)	Ballots (3)	Joint projects (2)	People's panel (6)
Announcements (7)	e‐Consult (3, 7)	Deliberative polling (3, 7)	Multistakeholder initiative (2)	Policy councils (1, 3)
Brochures (4–6)	Online feedback (4)	Discrete choice experiment (1)	Partnerships (2, 6)	Strategy groups (3)
Bulletins (2)	Online consultation (2, 6)	e‐Consult (7)	Policy roundtables (7)	Colead programmes (4, 8)
Educations programmes (3, 7)	Participation networks (8)	Focus groups (4)	Planning committees (3)	
Information delivery/feedback forums (3, 5)	Public hearings (7)	Participatory decision making (2, 8)	Reference groups (2, 6, 7)	
Newsletters (5, 7)	Seminars (4)	Surveys (4)	Scenario building (3)	
Patient‐reported outcomes (8)	Submissions (1)	Taskforces (3, 7)	Taskforce (1)	
Public notices (7)	Virtual town square (1)	Virtual town square (1)	Colead (5, 8)	
Public presentations (2, 6)				
Speeches (2, 6)				
Video (5)				
Virtual town square (1)				

### 3.4 Q.3) What are the implications of consumer engagement frameworks for CALD consumer engagement in the Australian healthcare system?

Nine of the 11 frameworks mentioned engagement with CALD consumers, explicitly recognising that CALD consumers face additional barriers to engagement.^45^, ^46^, ^47^, ^48^, ^49^, ^50^, ^51^, ^52^, ^53^ Five frameworks mentioned CALD consumers while describing the concepts of community and principles of diversity and inclusion, without any specific discussion for engagement other than the need for addressing language barriers.^49^, ^50^, ^51^, ^52^, ^53^ The remaining four frameworks discussed CALD engagement in more detail, outlining organisational prerequisites and opportunities to enhance engagement activities.^45^, ^46^, ^47^, ^48^ These frameworks were more recent, originating from 2015 onwards, with one framework originating at the federal level and three originating at the state level (New South Wales and Victoria), and highlighted different strategies going forward to address language barriers.

Organisational prerequisites to enhance opportunities for engagement for CALD consumers focused on three key initiatives: (1) provision of culturally sensitive services; (2) greater access to interpreters at the service level; and (3) inclusion of CALD consumers in committees. Provision of culturally sensitive services and engagement strategies was discussed in three frameworks^46^, ^47^, ^48^; however, no clear definition of culturally sensitive services was provided in these documents. External tools and resources were referred to in these frameworks to develop culturally sensitive services. Cultural diversity plans, strategic plans recognising the health needs of CALD consumers and committees representing CALD consumers were identified as mediums through which culturally sensitive services can be developed to optimise opportunities for engagement for CALD consumers.^47^, ^48^ The need for adequate financial and physical resources and dedicated time was identified as a prerequisite for all consumers; however, for CALD consumers, the need for increased access to interpreters was highlighted as an additional resource requirement in one framework.^46^ While identifying consumers from diverse backgrounds was discussed in the context of broader minority and priority groups overall, identifying CALD consumers was considered essential for advisory committees and governance activities.^45^ However, the degree to which diversity within CALD participants needs to be included in such committees was not clear. The mechanism to involve CALD consumers in advisory panels and committees was described in one framework,^45^ which proposed the recruitment of CALD consumers by multicultural health units or services within the organisation.^45^

Discussion of the facilitation of engagement activities to improve engagement with CALD consumers was focused on two components: (1) addressing language barriers and (2) training staff in cultural responsiveness. Addressing language barriers was discussed in two frameworks in the context of providing consumers with accredited interpreters where needed, where health information was prepared in relevant languages and formats, and policy was developed that mandates the use of accredited interpreters.^46^, ^47^ The provision of orientation and training for consumers was discussed as critical for effective engagement for all consumers; however, for CALD consumers, two frameworks emphasised providing training to staff and providers, which were designed to improve their cultural responsiveness.^46^, ^47^ This training was intended to create an environment that is culturally safe, and to ensure that language barriers are addressed when engaging with CALD consumers, thereby enhancing engagement activities.^46^, ^47^ However, there was limited explanation of what constitutes cultural responsiveness or culturally safe care. The policy mandating interpreter use and the recommendation for staff and provider training in cultural responsiveness appear to have been discussed in the context of improving engagement at a clinical care level rather than participating in healthcare decision making at the health service or organisational level.

## 4 DISCUSSION

Health services in Australia and internationally recognise not only the need for consumer engagement but also the need to demonstrate meaningful partnerships rather than tokenistic actions.^31^, ^39^, ^55^ The critical need to recognise minority and priority groups such as CALD consumers is also highlighted.^39^, ^55^ Our document analysis provides evidence of the need for this shift in the landscape of consumer engagement, and particularly the need for greater clarity regarding approaches to enhance minority representation and active participation. This analysis showed that while engagement was described as a process, conceptualisation of the process was limited.^45^, ^47^, ^48^ While many of the activities and methods used for engagement were described, there was a limited exploration of activities against the intended outcomes, with the evaluation of these activities limited to collecting feedback and surveys from consumers.^49^, ^52^ Addressing language barriers remains a key area of focus for enhancing engagement with CALD consumers, whereby recent frameworks acknowledged the role of culturally sensitive services and health professional cross‐cultural training to improve opportunities for engagement.^45^, ^46^, ^47^, ^48^

Our findings are similar to a recent analysis of migrant health policies in European countries, which demonstrated that policy initiatives for migrant health were largely focused on interpreter use and health professional cross‐cultural education.^56^ Culturally sensitive services were identified as a prerequisite to enhance CALD consumer engagement.^48^, ^53^ Cultural sensitivity refers to being aware of cultural diversity, including the influence of culture on consumers' values beliefs, and attitudes and acknowledging and respecting these differences^57^; yet, the lack of shared understanding of what constitutes a culturally sensitive service and how this can be achieved is an ongoing challenge.^58^, ^59^

The promotion of cultural competence at the system, organisation and staff levels was recognised as a critical prerequisite for a culturally sensitive service.^22^ Cultural competency in healthcare staff is described as the ability of staff to effectively deliver healthcare that meets the social, cultural and linguistic needs of patients.^22^ Our analysis identified that training healthcare staff in cultural responsiveness (capacity to respond to health issues of diverse communities) is crucial for enhancing activities of engagement with CALD consumers.^48^, ^60^ Cultural competency training is incorporated into health policy documents and directives,^61^, ^62^ patient safety and quality frameworks^63^ and professional accreditation standards^64^, ^65^ nationally and internationally. Wide variations exist between the conceptualisation of cultural competency training and how the training programmes are delivered, leading to variation in the outcomes.^66^ In Australia, health policy documents and health services have largely focused on cultural competency training for indigenous populations, with limited discussion and inclusion for CALD populations. Reliance on training alone without system‐ and organisation‐level changes may not be effective in developing culturally sensitive health services.^22^ Recent research also identified that cultural competency training programmes developed in partnership with local communities and tailored to meet the local population characteristics are essential for the success of such programmes, especially for minority consumers.^59^, ^67^ Creating cultural curiosity and the desire to learn about other cultures underpin the delivery of culturally competent care. Further understanding with regard to how to develop cultural curiosity is needed as a foundation for cultural competency training to promote engagement with CALD consumers.^22^

Meaningful engagement is considered to occur when both consumers and service providers have the necessary skills, knowledge and resources to support ongoing, reciprocal interaction.^25^, ^68^ Recognising this meaningfulness, most engagement frameworks in this document analysis proposed capacity building and training of consumers and organisational staff, dedicated time and physical and financial resources as essential elements to enhance opportunities for meaningful engagement.^47^, ^48^, ^50^, ^53^ These elements align with recent evidence that indicates that successful operationalization of engagement activities in healthcare is contingent upon organisational and individual capacity and motivation, institutional dynamics and resources for engagement.^30^

A key resource notable from across all frameworks is timely access to interpreters and translation, with the discussion of resources beyond those to address language barriers being limited.^46^, ^47^ State‐level health departments in Australia have a language services policy that dictates the mandatory use of interpreters for patients who are not fluent in English.^69^, ^70^ While these policies support the use of accredited interpreters for CALD consumers who are not fluent in English, their use is largely discussed as essential in the context of consent, decision making for medical and surgical treatments and in health services research.^70^ Evidence for use of interpreters in healthcare is also largely focused on engagement at the level of point of care, highlighting the importance of cultural and individual factors^71^ with the dearth of evidence on how interpreters are used and their effectiveness for CALD consumer engagement in high‐level decision making such as participation in safety and quality committees.

### 4.1 Implications

A broad range of activities has been proposed to promote consumer engagement at all levels of healthcare decision making, with some methods and activities, such as information displays, focus groups and workshops and participation in committees identified commonly across frameworks.^48^, ^72^ Despite the range of activities identified to enhance engagement, there is currently little consistent and clear information regarding the depth and type of engagement that may arise from a given activity; therefore, it is challenging for healthcare services to determine which engagement activity is best suited to achieve a particular purpose. Of key interest in the present study, there was little evidence regarding the involvement of CALD consumers in engagement activities and those activities that are suitable for CALD consumers or require additional support or adaptations to work effectively.

Our recent research with CALD consumer representatives across Australian healthcare settings highlighted that promoting flexible approaches to participation in consumer engagement activities is critical to enable participation from a diverse CALD population.^73^ Embedding flexibility for consumers that are convenient to their schedule but also recognise their health condition, caregiving and other responsibilities can promote an enduring and deeper relationship.^74^, ^75^ The need to value service user input through sufficient reimbursement is also recognised as important for all consumers but particularly those who may be from lower socioeconomic backgrounds, who live further from central city locations or who have caregiving responsibilities.^74^ Modelling policies and guidelines internationally on consumer reimbursement at the national level in Australia may provide one approach to ensure that sufficient reimbursement is consistently provided.^76^, ^77^

Given the range of CALD background and diversity between and within groups, the gold‐standard approach to ensuring sufficient and appropriate diversity for CALD consumer engagement activities remains elusive;^4^ the documents reviewed were noteworthy for their lack of discussion about the need to consider sociocultural diversity across CALD populations and how sufficiently diverse input might be determined. International literature also supports our findings for the lack of diverse minority representation in consumer engagement literature.^39^, ^78^ Guidelines for engaging with multicultural communities encourage collaboration with established multicultural services or multicultural committees to seek participants,^45^, ^79^ which are also recognised as a way to enhance consumer participation internationally.^80^ This approach may lead to disproportionate participation from some CALD groups or some individuals with the inadequate representation of new and emerging CALD groups.^56^ Over time, the use of well‐trained consumers in research may lead to desensitisation to the needs of their community.^81^ There currently appears to be limited consideration of nuanced sociocultural differences within and between populations.^23^ Consumer engagement frameworks provide a valuable opportunity to highlight some of these issues and drive thought and discussion regarding approaches to address issues of widening participation and diversity in CALD consumer engagement nationally and internationally.

Continuous improvement, as a key principle within healthcare, was prominent within the included frameworks. Evaluation of consumer involvement was discussed in many of the frameworks, but not its application for continuous improvement in the methods and activities used for consumer engagement and specifically for CALD groups. Integrating routine feedback and dissemination of lessons learned from consumer engagement activities within consumer engagement frameworks is the next step to support the dissemination of evidence‐based methods and activities across healthcare systems. Using a collaborative approach with consumers to evaluate the effectiveness of the consumer engagement methods and activities used and the adaptations or support required for CALD groups will contribute to ensuring that optimal methods and activities are used.^82^

### 4.2 Limitations

Our search across the national‐ and state‐level Australian health departments yielded a small sample of documents that fulfilled the inclusion criteria for the present analysis. A range of wider documents that refer or relate to consumer engagement including toolkits for engagement, strategic plans and diversity plans were not included in the present analysis, but may provide wider contextual information or specific support for the application of engagement strategies. Further work may extend the present analysis to explore where consumer engagement frameworks sit within broader guidelines and strategic or diversity plans or may explore the support available for specific activities through available toolkits. By limiting the analysis to the Australian health care setting, our findings are not generalisable outside of Australia, but in exploring frameworks that utilise internationally recognised models of engagement, the findings may have relevance beyond the national context. A comparative analysis is required. Our analysis also focused on the implications of consumer engagement frameworks for CALD populations; consumer engagement for other minority and priority populations requires exploration.

## 5 CONCLUSION

While the engagement frameworks established the need for effective consumer engagement, discussion of the mechanisms to achieve this goal for CALD groups was limited. Addressing language barriers by using interpreters and translated resources was the key focus, with recent frameworks emphasising the need for culturally sensitive services to improve engagement with CALD consumers. However, discussion of what culturally sensitive services look like and what resources are needed to improve engagement with CALD consumers in high‐level decision making in health care were lacking. There is currently little consistent and clear information regarding the depth and type of engagement that may arise from a given engagement activity, especially for CALD consumers. Evaluating and adapting the activities in collaboration with CALD consumers can enhance the effectiveness of the activities of engagement. A flexible approach to participation and developing mechanisms for reimbursement for participation may enhance engagement with CALD consumers. The engagement frameworks present scope to develop approaches to widening participation and diversity in CALD consumer engagement.

## CONFLICT OF INTERESTS

The authors declare that there are no conflicts of interest.

## AUTHOR CONTRIBUTIONS

Ashfaq Chauhan, Reema Harrison and Ramesh L. Walpola conceptualised the study. Ashfaq Chauhan and Jiadai Li completed data collection and extraction. Ashfaq Chauhan completed the data analysis and draft of the manuscript. All authors discussed the draft and provided feedback. All authors approved the final version for submission.

## Supporting information

Supporting information.

Supporting information.

Supporting information.

## Data Availability

The data that support the findings of this study are available from the corresponding author upon reasonable request.
