# Supplementary material for: How do health services engage culturally and linguistically diverse consumers? An analysis of consumer engagement frameworks in Australia
Source: Health Expect. 2021 Jul 15;24(5):1747–62. doi: 10.1111/hex.13315 (PMC8483202; doi:10.1111/hex.13315)
Supplement: Supplementary file 2 — Supporting information. [file HEX-24-1747-s001.docx]

Supplementary File B

1. Australian Government Department of Health.
2. New South Wales (NSW) Health.
3. Department of Health and Human Services, Victoria (VIC).
4. South Australia Health.
5. Department of Health, Western Australia.
6. Department of Health, Queensland.
7. Department of Health, Northern Territory.
8. ACT Health, Australian Capital Territory.
9. Department of Health, Tasmania.
10. Better Care, Victoria.
11. Safer Care, Victoria.
12. Agency for Clinical Innovation (ACI), NSW.
13. Clinical Excellence Commission (CEC), NSW.
14. Cancer Institute, NSW.
15. Cancer Australia
16. Aged Care Quality and Safety Commission
17. Australian Commission on Safety and Quality in Health Care
18. Australian Digital Health Agency
19. Australian Institute of Health and Welfare
20. National Health and Medical Research Council
