# Supplementary material for: How do health services engage culturally and linguistically diverse consumers? An analysis of consumer engagement frameworks in Australia
Source: Health Expect. 2021 Jul 15;24(5):1747–62. doi: 10.1111/hex.13315 (PMC8483202; doi:10.1111/hex.13315)
Supplement: Supplementary file 3 — Supporting information. [file HEX-24-1747-s002.docx]

**Supplementary File C: Common characteristics and differences between the frameworks.**

| **Characteristics** | **Frameworks** | | | | | | | | | | |
| --- | --- | --- | --- | --- | --- | --- | --- | --- | --- | --- | --- |
|  | **CI NSW. New South Wales** | **DHHS Tasmania** | **ACI, New South Wales** | **Safer Care, VIC** | **DHHS, VIC** | **DoH, Australia** | **DoH, Queensland** | **DoH, Northern Territory** | **SA Health, South Australia** | **WA Health, Western Australia** | **Cancer Australia** |
| **Year** | 2015 | 2014 | 2015 | 2019 | 2019 | 2017 | 2012 | 2012 | 2013 (updated 2017) | 2007 | 2011 |
| **Explicit definition of engagement** | No | Yes | Yes | No | Yes | Yes | Yes | Yes | Yes | Yes | Yes |
| **Principles of engagement** | No | Yes | No | No | Yes | Yes | Yes | Yes | Yes | Yes | Yes |
| **Conceptualisation of the process of engagement** | No | No | Yes | No | Yes | Yes | No | No | No | No | No |
| **Continuum of engagement** | Yes | Yes (discussed as participation continuum) | Yes (Discussed as level) | No | Yes  (discussed as spectrum) | Yes (discussed as levels) | Yes (discussed as elements) | Yes (discussed as spectrum) | No | Yes  (discussed as degree of control) | Yes |
| **Methods or activities of engagement** | Yes | Yes | Yes | No | Yes | Yes | Yes | Yes | No | No | Yes |
| **Evaluation discussed** | No | Yes | Yes | Yes | Yes | Yes | Yes | Yes | Yes | Yes | Yes |
| **Minority and priority population mentioned (Diversity)** | Yes | Yes | Yes | Yes | Yes | Yes | Yes | Yes | Yes | Yes | Yes |
| **CALD population mentioned separately** | No | No | Yes | Yes | Yes | Yes | Yes (in examples provided) | Yes | Yes | Yes (language barrier discussed but no other specific discussion) | No |
| **Specific strategies for CALD consumer engagement discussed** | No | No | Yes | Yes | Yes | Yes | No | No | No | No | Yes |
